# Occupation and occurrence of respiratory infections among adults with newly diagnosed asthma

**DOI:** 10.1186/s12890-023-02413-8

**Published:** 2023-04-25

**Authors:** Maritta S. Jaakkola, Taina K. Lajunen, Aino K. Rantala, Rachel Nadif, Jouni J. K. Jaakkola

**Affiliations:** 1 Center for Environmental and Respiratory Health Research, and Biocenter, University of Oulu, Oulu, Finland; 2 Center for Environmental and Respiratory Health Research, University of Oulu, Aapistie 5B, P.O.Box 5000, 90014 Oulu, Finland; 3 Université Paris-Saclay, UVSQ, Univ. Paris-Sud, INSERM, Equipe d’Epidémiologie Respiratoire Intégrative, CESP, 94807 Villejuif, France; 4 Finnish Meteorological Institute, Erik Palménin Aukio 1, 00560 Helsinki, Finland

**Keywords:** Respiratory infections, Asthma, Occupation, Spreading

## Abstract

**Background:**

Work environments are potential areas for spreading respiratory infections. We hypothesized that certain occupations increase susceptibility to respiratory infections among adults with asthma. Our objective was to compare the occurrence of respiratory infections among different occupations in adults with newly diagnosed asthma.

**Methods:**

We analysed a study population of 492 working-age adults with newly diagnosed asthma who were living in the geographically defined Pirkanmaa Area in Southern Finland during a population-based Finnish Environment and Asthma Study (FEAS). The determinant of interest was occupation at the time of diagnosis of asthma. We assessed potential relations between occupation and occurrence of both upper and lower respiratory tract infections during the past 12 months. The measures of effect were incidence rate ratio (IRR) and risk ratio (RR) adjusted for age, gender, and smoking habits. Professionals, clerks, and administrative personnel formed the reference group.

**Results:**

The mean number of common colds in the study population was 1.85 (95% CI 1.70, 2.00) infections in the last 12 months. The following occupational groups showed increased risk of common colds: forestry and related workers (aIRR 2.20, 95% CI 1.15–4.23) and construction and mining (aIRR 1.67, 95% CI 1.14–2.44). The risk of lower respiratory tract infections was increased in the following groups: glass, ceramic, and mineral workers (aRR 3.82, 95% CI 2.54–5.74), fur and leather workers (aRR 2.06, 95% CI 1.01–4.20) and metal workers (aRR 1.80, 95% CI 1.04–3.10).

**Conclusions:**

We provide evidence that the occurrence of respiratory infections is related to certain occupations.

**Supplementary Information:**

The online version contains supplementary material available at 10.1186/s12890-023-02413-8.

## Background

Respiratory infections are relatively common in working-age adults and lead often to absence from work. Acute respiratory infections may exacerbate existing bronchial asthma, while recurrent respiratory infections may have adverse consequences for long-term prognosis of asthmatics [1, 2]. We have previously reported that sharing the office is a risk factor for increased occurrence of respiratory infections among office workers, but otherwise office work environment is considered to include less risk factors for respiratory health compared to many other occupations [3].

The current Covid-19 pandemic raised our interest in studying if we can identify occupations that are related to increased risk of having acute respiratory infections. Such information would be useful for guiding preventive actions to control spreading of respiratory infections at work.

The objective of the present study was to compare the occurrence of respiratory infections among different occupations in working-age adults with newly diagnosed asthma. Those with asthma are likely to form a group especially susceptible to respiratory infections [4, 5]. Professionals, clerks, and administrative personnel formed the reference category in this study.

## Methods

### Study design and study population

This study was based on a population-based case–control study of incident asthma, the Finnish Environment and Asthma Study (FEAS) [6, 7]. In the present study, the study population included 492 working-age adults with incident asthma (94.4% of all cases in FEAS), who had all the information needed for determining occurrence of respiratory infections in each occupational group at the time of diagnosis of asthma.

The ethics committees of the Pirkanmaa Hospital District and the Finnish Institute of Occupational Health had approved the study.

New cases of asthma were recruited at all health-care facilities diagnosing asthma in the Pirkanmaa District in Southern Finland, including the Tampere University Hospital, public health care centers and centers of private practicing pulmonary physicians. In addition, the National Social Insurance Institution of Finland invited to participate all patients 21–63 years old, who had received new reimbursement right for asthma medication in this area during the study period, but who had not yet participated [7]. Altogether 521 cases of new adult-onset asthma participated in the FEAS, with total participation rate of 86%. Among those excluded from the present study population, 17 subjects had not reported occupation at the time of asthma diagnosis, 10 had reported no information on infections, and 2 had reported neither occupation nor infections at the time of asthma diagnosis. Thus, these results are based on 492 working-age adults with adult-onset asthma, i.e. 94.4% of all cases in FEAS.

The following criteria were applied for defining asthma in FEAS: 1) reporting at least one asthma-related symptom, i.e. prolonged cough, wheezing, attacks of or exercise-induced dyspnea, and/or nocturnal cough or wheezing, and 2) demonstration of reversibility in airways obstruction in lung function investigations [6]. These criteria were compatible with the criteria that were required for the diagnosis of asthma in Finland at the time of the study recruitment period.

### Data collection

The study subjects answered a self-administered questionnaire modified from the Helsinki Office Environment Study questionnaire for use in a general population [6]. The questionnaire included six sections: 1) personal characteristics, 2) health information, 3) active smoking and exposure to environmental tobacco smoke, 4) occupation and work environment, 5) home environment, and 6) dietary questions. The section on occupation and work environment inquired about current occupation, followed by previous occupations throughout the subject’s working history, recalling occupational history backwards. Additional questions inquired about some details of the current indoor environment at work (at the time of asthma diagnosis). The subjects were also asked whether they had changed their job because of respiratory problems and, in the case of a confirmatory answer, to indicate the job that was in question and to describe the symptoms.

### Occupation as the determinant of interest

The determinant of interest was occupation at the time of the diagnosis of asthma. We applied the International Standard Classification of Occupations-88 to code the reported occupations, and then identified 25 groups of occupations. The reference category included professionals, clerks, and administrative personnel. To classify each subject into an occupational group, we used current job at the time of asthma diagnosis or up to 3 months prior to it, or the most recent occupation if the subject had quit work because of respiratory symptoms related to bronchial asthma. If the subject had quit work because of respiratory symptoms, the occupation which the subject had quit was used as the determinant.

### Respiratory infections as the outcomes

The main outcomes of interest were respiratory infections during the past 12 months. Assessment of occurrence of respiratory infections was based on the following question in the study questionnaire: *How often did you experience the following infections during the past 12 months?* The list of infections included common cold, tonsillitis, sinusitis, otitis media, acute bronchitis, and pneumonia. Respiratory infections were classified as upper respiratory tract infections (URTI), including common cold, tonsillitis, sinusitis and otitis media, and as lower respiratory tract infections (LRTI), including acute bronchitis and pneumonia. We applied two measures of occurrence: 1) incidence rate (IR) of common cold and URTIs, and 2) the risk (R) of tonsillitis, sinusitis and otitis media, and among lower respiratory tract infections (LRTI), the risk of acute bronchitis and pneumonia.

### Statistical methods

We applied incidence rate per person-year (IR) and risk (R) during the past 12 months as the measures of occurrence of the respiratory infections. We estimated the occupation as the determinant of respiratory infections by calculating incidence rate ratios (IRR) and risk ratios (RR) of infections as the measures of effect. The reference category in these comparisons was professionals, clerks, and administrative personnel. We adjusted primarily for age, sex and personal smoking (core covariates). In addition, we conducted sensitivity analyses of URTI and LRTI by adjusting for environmental tobacco smoke and dampness and molds at home and for having children and finally including the core covariates and all the three additional covariates in the model (saturated model). For estimation of IRRs we used the SAS Proc genmod with Poisson distribution and log link, and for estimation of mean IRs we used the Proc Means in SAS statistical package version 9.4 (SAS Institute Inc., Cary, NC). We estimated the effect of occupation on the occurrence of LRTI by calculating RR (risk ratio), using the SAS Proc genmod with Poisson distribution and log link, applying Zou’s modified Poisson regression [8].

## Results

The study subjects of the FEAS were 20–63 years old and the distribution of smoking habits among the asthma population in the present analyses was the following: Never smokers 50.0%, Former smokers 22.7%, and Current smokers (including both regular and occasional smokers) 27.3%. Furthermore, 22.5% reported ETS in the workplace and/or at home. Because the majority of the study subjects were recruited before the asthma diagnosis was verified in the clinical investigations (n = 362, 69.5%), they could have as medication short-acting bronchodilator as needed, but no long-term medication for treating the inflammation of the airways, i.e. no inhaled steroids or regular oral corticosteroid. The incidence rate (95% confidence interval) of common colds among this working-age population was 1.85 (95% CI 1.73, 1.97) per 12 months (Table 1). In the reference occupational group of professionals, clerks, and administrative personnel the average incidence rate in 12 months was 1.70 (1.41, 1.99), i.e. slightly lower.

**Table 1:** Numbers of upper and lower respiratory tract infections during past 12 months in people with newly-onset adult asthma according to the occupation in the FEAS

Occupation	Total N^a^	Common cold mean number (95% CI)^a^	Common cold n (%)^a,^^b^	Tonsillitis n (%)^a,^^b^	Sinusitis n (%)^a,^^b^	Otitis media n (%)^a,^^b^	URTI n (%)^a,^^b^	Acute bronchitis n (%)^a,^^b^	Pneumonia n (%)^a,^^b^	LRTI n (%)^a,^^b^
2 Bakers and food processors	7	2.00 (0.0, 4.33)	4 (57.1)	0 (0.0)	1 (14.3)	3 (42.9)	3 (42.9)	1 (14.3)	1 (14.3)	1 (14.3)
3 Chemical industry workers	4	2.25 (0.0, 5.26)	4 (100.0)	0 (0.0)	1 (25.0)	0 (0.0)	1 (25.0)	2 (50.0)	0 (0.0)	2 (50.0)
4 Cleaners	31	1.45 (0.82, 2.08)	18 (58.1)	2 (6.5)	12 (38.7)	1 (3.2)	13 (41.9)	13 (41.9)	2 (6.5)	13 (41.9)
5 Construction and mining workers	13	2.54 (1.29, 3.79)	11 (84.6)	0 (0.0)	4 (30.8)	2 (15.4)	5 (38.5)	4 (30.8)	1 (7.7)	4 (30.8)
6 Day-care workers	10	1.70 (0.27, 3.13)	6 (60.0)	2 (20.0)	5 (50.0)	1 (10.0)	6 (60.0)	3 (30.0)	0 (0.0)	3 (30.0)
7 Dentists and dental workers	3	0.67 (0.0, 3.54)	1 (33.3)	0 (0.0)	1 (33.3)	0 (0.0)	1 (33.3)	1 (33.3)	0 (0.0)	1 (33.3)
8 Drivers	6	1.50 (0.0, 3.09)	4 (66.7)	0 (0.0)	2 (33.3)	0 (0.0)	2 (33.3)	3 (50.0)	1 (16.7)	3 (50.0)
9 Electrical and electronic production workers	8	1.25 (0.09, 2.41)	4 (50.0)	0 (0.0)	2 (25.0)	0 (0.0)	2 (25.0)	2 (25.0)	0 (0.0)	2 (25.0)
10 Engine workshop workers	7	1.71 (0.83, 2.59)	6 (85.7)	1 (14.3)	3 (42.9)	0 (0.0)	3 (42.9)	2 (28.6)	0 (0.0)	2 (28.6)
11 Farmers and agricultural workers	15	1.93 (0.94, 2.93)	12 (80.0)	2 (13.3)	5 (33.3)	0 (0.0)	6 (40.0)	0 (0.0)	2 (13.3)	2 (13.3)
12 Forestry and related workers	3	3.33 (0.0, 15.59)	2 (66.7)	0 (0.0)	0 (0.0)	0 (0.0)	0 (0.0)	0 (0.0)	1 (33.3)	1 (33.3)
13 Fur and leather workers	4	1.25 (0.0, 3.64)	2 (50.0)	1 (25.0)	3 (75.0)	1 (25.0)	3 (75.0)	2 (50.0)	1 (25.0)	3 (75.0)
14 Glass, ceramic, and mineral workers	2	0.50 (0.0, 6.85)	1 (50.0)	0 (0.0)	0 (0.0)	0 (0.0)	0 (0.0)	2 (100.0)	0 (0.0)	2 (100.0)
15 Hairdressers	5	2.20 (0.58, 3.82)	5 (100.0)	2 (40.0)	3 (60.0)	0 (0.0)	3 (60.0)	1 (20.0)	0 (0.0)	1 (20.0)
16 Housewives	5	3.20 (1.36, 5.04)	5 (100.0)	0 (0.0)	1 (20.0)	1 (20.0)	2 (40.0)	1 (20.0)	0 (0.0)	1 (20.0)
17 Laboratory technicians	2	2.50 (0.0, 8.85)	2 (100.0)	1 (50.0)	2 (100.0)	0 (0.0)	2 (100.0)	1 (50.0)	1 (50.0)	1 (50.0)
18 Metal workers	25	2.00 (1.32, 2.68)	20 (80.0)	1 (4.0)	5 (20.0)	0 (0.0)	6 (24.0)	12 (48.0)	1 (4.0)	12 (48.0)
19 Maternity leave	1	3.00 (NA)	1 (100.0)	1 (100.0)	0 (0.0)	1 (100.0)	1 (100.0)	0 (0.0)	0 (0.0)	0 (0.0)
20 Nurses and nursing associates	27	2.15 (1.51, 2.79)	22 (81.5)	2 (7.4)	10 (37.0)	0 (0.0)	10 (37.0)	7 (25.9)	1 (3.7)	7 (25.9)
21 Painters	2	2.00 (0.0, 14.7)	2 (100.0)	0 (0.0)	0 (0.0)	0 (0.0)	0 (0.0)	0 (0.0)	0 (0.0)	0 (0.0)
22 Physicians	2	1.00 (NA)	2 (100.0)	0 (0.0)	0 (0.0)	0 (0.0)	0 (0.0)	1 (50.0)	0 (0.0)	1 (50.0)
23 Printers	2	1.50 (0.0, 7.85)	2 (100.0)	0 (0.0)	1 (50.0)	0 (0.0)	1 (50.0)	0 (0.0)	0 (0.0)	0 (0.0)
24 Rubber and plastic workers	8	1.38 (0.49, 2.26)	6 (75.0)	0 (0.0)	2 (25.0)	0 (0.0)	2 (25.0)	1 (12.5)	0 (0.0)	1 (12.5)
25 Retired	39	1.36 (0.89, 1.83)	27 (69.2)	2 (5.1)	10 (25.6)	4 (10.3)	14 (35.9)	19 (48.7)	1 (2.6)	20 (51.3)
26 Sick leave	1	1.00 (NA)	1 (100.0)	0 (0.0)	0 (0.0)	0 (0.0)	0 (0.0)	0 (0.0)	0 (0.0)	0 (0.0)
27 Storage workers	7	1.57 (0.0, 3.25)	5 (71.4)	0 (0.0)	0 (0.0)	1 (14.3)	1 (14.3)	1 (14.3)	0 (0.0)	1 (14.3)
28 Students	30	2.47 (1.86, 3.08)	27 (90.0)	2 (6.7)	8 (26.7)	2 (6.7)	12 (40.0)	9 (30.0)	1 (3.3)	9 (30.0)
29 Textile workers	9	2.11 (0.99, 3.23)	8 (88.9)	0 (0.0)	1 (11.1)	0 (0.0)	1 (11.1)	2 (22.2)	0 (0.0)	2 (22.2)
30 Other occupations	38	2.00 (1.31, 2.69)	29 (76.3)	3 (7.9)	10 (26.3)	5 (13.2)	12 (31.6)	17 (44.7)	0 (0.0)	17 (44.7)
31 Unemployed	24	2.42 (1.54, 3.30)	22 (91.7)	1 (4.2)	6 (25.0)	4 (16.7)	8 (33.3)	7 (29.2)	1 (4.2)	7 (29.2)
32 Waiters	13	2.00 (0.98, 3.02)	10 (76.9)	1 (7.7)	4 (30.8)	1 (0.2)	5 (38.5)	5 (38.5)	0 (0.0)	5 (38.5)
33 Wood and paper workers	9	1.44 (0.67, 2.22)	7 (77.8)	0 (0.0)	1 (11.1)	1 (11.1)	2 (22.2)	2 (22.2)	0 (0.0)	2 (22.2)
1 Professionals, clerks, and administrative (REFERENCE)	130	1.70 (1.41, 1.99)	99 (76.2)	11 (8.5)	35 (26.9)	10 (7.7)	41 (31.5)	49 (37.7)	4 (3.1)	50 (38.5)
TOTAL	492	1.85 (1.70, 2.00)	377 (76.6)	35 (7.11)	138 (28.1)	38 (7.7)	168 (34.2)	170 (34.6)	19 (3.9)	176 (35.8)
Occupation missing	17	2.06 (1.20, 2.92)	14 (82.4)	1 (5.9)	2 (11.8)	1 (5.9)	4 (25.5)	6 (35.3)	1 (5.9)	6 (35.3)

*Abbreviations*: *FEAS* Finnish Environment and Asthma Study, *LRTI* Lower respiratory tract infection, *URTI* Upper respiratory tract infection

^a^Information on infections missing for altogether 12 participants: 3 professionals, clerks and administrative, 1 chemical industry worker, 1 cleaner, 2 metal workers, 2 retired, 1 unemployed, 2 with occupation missing

^b^Number of participants with at least one infection

### Upper respiratory tract infections (URTI)

The following occupational groups showed increased risk of URTIs compared to the reference group (Table 2): Fur and leather workers with adjusted risk ratio (aRR) 2.87 (95% CI 1.49–5.54), laboratory technicians with aRR 2.87 (1.92–4.30) and hairdressers with aRR 2.14 (1.13–4.04). In addition, those on maternity leave reported increased number of URTI with aRR 2.41 (1.79–3.24). Furthermore, day-care workers showed increased crude RR of URTI 1.90 (1.08–3.35), but adjusted RR of 1.68 was not statistically significant (95% CI 0.96–2.94).

**Table 2:** Risk of upper respiratory tract infections during past 12 months in people with newly-onset adult asthma according to the occupation in the FEAS

	Common cold^a^		Tonsillitis^a,c,d^		Sinusitis^a,c,d^		Otitis media^a,c,d^		URTI^a,c,d,e^	
Occupation	Crude IRR (95% CI)	Adjusted^b^ IRR (95% CI)	Crude RR (95% CI)	Adjusted^b^ RR (95% CI)	Crude RR (95% CI)	Adjusted^b^ RR (95% CI)	Crude RR (95% CI)	Adjusted ^b^ RR (95% CI)	Crude RR (95% CI)	Adjusted^b^ RR (95% CI)
2 Bakers and food processors	1.18 (0.69–2.02)	1.06 (0.61–1.84)	NA	NA	0.53 (0.08–3.33)	0.62 (0.11–3.38)	**5.57 (1.96–15.80)**	**4.67 (1.45–15.05)**	1.36 (0.56–3.32)	1.46 (0.59–3.65)
3 Chemical industry workers	1.32 (0.68–2.58)	1.16 (0.60–2.27)	NA	NA	0.93 (0.17–5.19)	0.80 (0.15–4.15)	NA	NA	0.79 (0.14–4.41)	0.69 (0.13–3.57)
4 Cleaners	0.85 (0.62–1.18)	0.82 (0.59–1.14)	0.76 (0.18–3.27)	0.81 (0.19–3.49)	1.44 (0.85–2.43)	1.30 (0.78–2.17)	0.42 (0.06–3.15)	0.38 (0.05–2.89)	1.33 (0.82–2.16)	1.26 (0.78–2.04)
5 Construction and mining workers	**1.49 (1.04–2.15)**	**1.67 (1.14–2.44)**	NA	NA	1.14 (0.48–2.71)	1.84 (0.75–4.54)	2.00 (0.49–8.17)	2.53 (0.64–9.91)	1.22 (0.59–2.54)	1.67 (0.78–3.56)
6 Day-care workers	1.00 (0.61–1.64)	0.93 (0.56–1.52)	2.36 (0.61–9.23)	2.26 (0.63 -8.20)	1.86 (0.94–3.67)	1.57 (0.81–3.03)	1.30 (0.18–9.16)	1.14 (0.14–9.29)	**1.90 (1.08–3.35)**	1.68 (0.96–2.94)
7 Dentists and dental workers	0.39 (0.10–1.58)	0.38 (0.09–1.52)	NA	NA	1.24 (0.24–6.29)	0.997 (0.23–4.27)	NA	NA	1.06 (0.21–5.34)	0.95 (0.19–4.71)
8 Drivers	0.88 (0.45–1.72)	1.11 (0.56–2.17)	NA	NA	1.24 (0.39–3.98)	1.93 (0.51–7.30)	NA	NA	1.06 (0.33–3.37)	1.38 (0.39–4.80)
9 Electrical and electronic production workers	0.74 (0.39–1.39)	0.86 (0.46–1.64)	NA	NA	0.93 (0.27–3.19)	1.31 (0.41–4.18)	NA	NA	0.79 (0.23–2.70)	0.98 (0.30–3.25)
10 Engine workshop workers	1.01 (0.56–1.80)	0.99 (0.54–1.78)	1.69 (0.25–11.29)	1.13 (0.17–7.46)	1.59 (0.65–3.92)	2.34 (0.96–5.68)	NA	NA	1.36 (0.56–3.32)	1.52 (0.63–3.66)
11 Farmers and agricultural workers	1.14 (0.77–1.67)	1.31 (0.88–1.93)	1.58 (0.39–6.45)	2.06 (0.47–8.96)	1.24 (0.57–2.67)	1.40 (0.67–2.93)	NA	NA	1.27 (0.65–2.48)	1.38 (0.70–2.71)
12 Forestry and related workers	**1.96 (1.04–3.70)**	**2.20 (1.15–4.23)**	NA	NA	NA	NA	NA	NA	NA	NA
13 Fur and leather workers	0.74 (0.30–1.78)	0.87 (0.36–2.13)	2.95 (0.49–17.68)	4.44 (0.90–21.88)	**2.79 (1.48–5.24)**	**3.48 (1.76–6.87)**	3.25 (0.54–19.64)	4.30 (0.53–35.02)	**2.38 (1.28–4.42)**	**2.87 (1.49–5.54)**
14 Glass, ceramic, and mineral workers	0.29 (0.04–2.10)	0.29 (0.04–2.09)	NA	NA	NA	NA	NA	NA	NA	NA
15 Hairdressers	1.29 (0.71–2.37)	1.26 (0.66–2.38)	**4.73 (1.41–15.91)**	**6.23 (1.43–27.22)**	**2.22 (1.03–4.81)**	**2.22 (1.12–4.39)**	NA	NA	1.90 (0.89–4.06)	**2.14 (1.13–4.04)**
16 Housewives	1.88 (1.13–3.13)	1.35 (0.80–2.29)	NA	NA	0.74 (0.13–4.39)	0.61 (0.10–3.74)	2.60 (0.41–16.56)	1.40 (0.22–8.75)	1.27 (0.42–3.82)	1.02 (0.33–3.10)
17 Laboratory technicians	1.47 (0.61–3.57)	1.51 (0.62–3.68)	**5.91 (1.32–26.40)**	**5.23 (1.15–23.74)**	**3.71 (2.80–4.93)**	**3.74 (2.03–6.91)**	NA	NA	**3.17 (2.46 4.08)**	**2.87 (1.92–4.30)**
18 Metal workers	1.18 (0.87–1.60)	1.37 (0.99 -1.91)	0.47 (0.06–3.50)	0.42 (0.05–3.38)	0.74 (0.32–1.71)	1.23 (0.51–3.01)	NA	NA	0.76 (0.36–1.60)	0.98 (0.45–2.14)
19 Maternity leave	1.76 (0.56–5.51)	1.59 (0.51–4.99)	**11.82 (6.71–20.80)**	**11.22 (4.67–26.91)**	NA	NA	**13.00 (7.17–23.58)**	**10.23 (4.78–21.87)**	**3.17 (2.46–4.08)**	**2.41 (1.79–3.24)**
20 Nurses and nursing associates	1.26 (0.95–1.69)	1.08 (0.81–1.45)	0.88 (0.21–3.73)	0.79 (0.19–3.27)	1.38 (0.78–2.43)	1.12 (0.63–2.01)	NA	NA	1.17 (0.68–2.04)	1.03 (0.59–1.81)
21 Painters	1.18 (0.44–3.16)	1.16 (0.43–3.17)	NA	NA	NA	NA	NA	NA	NA	NA
22 Physicians	0.59 (0.15–2.37)	0.79 (0.20–3.21)	NA	NA	NA	NA	NA	NA	NA	NA
23 Printers	0.88 (0.28–2.76)	0.87 (0.28–2.73)	NA	NA	1.86 (0.45–7.64)	1.80 (0.28–11.35)	NA	NA	1.59 (0.39–6.49)	1.51 (0.26–8.76)
24 Rubber and plastic workers	0.81 (0.44–1.48)	0.89 (0.48–1.63)	NA	NA	0.93 (0.27–3.19)	1.10 (0.33–3.67)	NA	NA	0.79 (0.23–2.70)	0.86 (0.26–2.86)
25 Retired	0.80 (0.59–1.08)	1.00 (0.72–1.38)	0.61 (0.14–2.62)	1.09 (0.22–5.24)	0.95 (0.52–1.74)	1.22 (0.65–2.31)	1.33 (0.44–4.02)	1.86 (0.54–6.40)	1.14 (0.70–1.86)	1.45 (0.86–2.45)
26 Sick leave	0.59 (0.08–4.19)	0.93 (0.13–6.68)	NA	NA	NA	NA	NA	NA	NA	NA
27 Storage workers	0.92 (0.50–1.69)	1.03 (0.56–1.88)	NA	NA	NA	NA	1.86 (0.28–12.54)	2.05 (0.29–14.66)	0.45 (0.07–2.83)	0.47 (0.07–3.09)
28 Students	**1.45 (1.12–1.89)**	1.09 (0.81–1.47)	0.79 (0.18–3.37)	0.49 (0.11–2.19)	0.99 (0.51–1.91)	1.02 (0.46–2.22)	0.87 (0.20–3.75)	0.51 (0.11–2.33)	1.27 (0.76–2.10)	1.17 (0.65–2.10)
29 Textile workers	1.24 (0.78–1.98)	1.17 (0.73–1.88)	NA	NA	0.41 (0.06–2.68)	0.41 (0.06–2.71)	NA	NA	0.35 (0.06–2.27)	0.36 (0.05–2.47)
30 Other occupations	1.18 (0.91–1.53)	1.16 (0.89–1.51)	0.93 (0.27–3.17)	0.92 (0.28–3.07)	0.98 (0.54–1.79)	1.01 (0.57–1.79)	1.71 (0.62–4.70)	1.62 (0.59–4.40)	1.00 (0.59–1.70)	1.02 (0.61–1.72)
31 Unemployed	**1.42 (1.06–1.90)**	1.28 (0.95–1.73)	0.49 (0.07–3.64)	0.40 (0.05–3.00)	0.93 (0.44–1.96)	0.98 (0.48–1.99)	2.17 (0.74–6.35)	1.85 (0.68–5.07)	1.06 (0.57–1.96)	1.07 (0.59–1.96)
32 Waiters	1.18 (0.78–1.77)	0.92 (0.61–1.39)	0.91 (0.13–6.49)	0.69 (0.11–4.45)	1.14 (0.48–2.71)	0.87 (0.37–2.08)	1.00 (0.14–7.21)	0.68 (0.09–5.29)	1.22 (0.59–2.54)	0.99 (0.47–2.08)
33 Wood and paper workers	0.85 (0.49–1.49)	0.84 (0.48–1.48)	NA	NA	0.41 (0.06–2.68)	0.51 (0.07–3.59)	1.44 (0.21–10.07)	1.42 (0.23–8.82)	0.70 (0.20–2.45)	0.77 (0.22–2.73)
Professionals, clerks, and administrative (REFERENCE)	REF	REF	REF	REF	REF	REF	REF	REF	REF	REF

*Abbreviations*: *CI* Confidence interval, *IRR* Incidence rate ratio, *RR* Risk ratio, *URTI* Upper respiratory tract infection

^a^Information on infections missing for altogether 12 participants: 3 professionals, clerks and administrative, 1 chemical industry worker, 1 cleaner, 2 metal workers, 2 retired, 1 unemployed, 2 with occupation missing

^b^Adjusted for sex, age, and smoking

^c^Estimate for having at least one infection

^d^Participants with occupations that could not be included in the model (NA) were excluded from the analysis

Among the URTIs, the IRR of common colds was increased among forestry and related workers (aIRR 2.20, 95% CI 1.15–4.23) and construction and mining workers (aIRR 1.67, 95% CI 1.14–2.44) (Table 2). In addition, crude IRR of common colds was also significantly increased among students (IRR 1.45, 95% CI 1.12–1.89) and the unemployed (1.42, 1.06–1.90). However their lower confidence interval included 1 when adjusted for sex, age, and smoking. Occurrence of tonsillitis was increased especially among hairdressers (aIRR 6.23, 1.43–27.22), laboratory technicians (5.23, 1.15–23.74) and among those on maternity leave (11.22, 4.67–26.91). Occurrence of sinusitis was increased among fur and leather workers (aRR 3.48, 95% CI 1.76–6.87), hairdressers (2.22, 1.12–4.39), and among laboratory technicians (3.74, 2.03–6.91). And finally, increased occurrence of otitis media was detected among bakers and food processors (aRR 4.67, 95% CI 1.45–15.05) and among those on maternity leave (10.23, 4.78–21.87).

### Lower respiratory tract infections (LRTI)

The risk of LRTIs was increased in the following occupations: glass, ceramic, and mineral workers (aRR 3.82, 95% CI 2.54–5.74), fur and leather workers (2.06, 95% CI 1.01–4.20), and metal workers (1.80, 95% CI 1.04–3.10) (Table 3). When analyzing LRTIs separately, the risk of acute bronchitis was increased among glass, ceramic, and mineral workers (3.96, 2.61–6.01) and metal workers (1.84, 1.06–3.18). The risk of pneumonia was increased among laboratory workers (30.66, 6.55–143.48), forestry and related workers (19.79, 3.23–121.32), and among drivers (9.48, 1.14 -78.92).

**Table 3:** Risk of lower respiratory tract infections during past 12 months in people with newly-onset adult asthma according to the occupation in the FEAS

	Acute bronchitis^a,b,c^		Pneumonia^a,b,c^		LRTI^a,b,c^	
Occupation	Crude RR (95% CI)	Adjusted^d^ RR (95% CI)	Crude RR (95% CI)	Adjusted^d^ RR (95% CI)	Crude RR (95% CI)	Adjusted^d^ RR (95% CI)
2 Bakers and food processors	0.38 (0.06–2.36)	0.43 (0.07–2.70)	4.64 (0.59–36.25)	4.35 (0.51–37.16)	0.37 (0.06–2.31)	0.41 (0.06–2.59)
3 Chemical industry workers	1.33 (0.49–3.62)	1.22 (0.51–2.93)	NA	NA	1.30 (0.48–3.55)	1.21 (0.50–2.93)
4 Cleaners	1.11 (0.70–1.78)	0.98 (0.61–1.57)	2.10 (0.40–10.93)	1.42 (0.28–7.09)	1.09 (0.68–1.74)	0.96 (0.60–1.54)
5 Construction and mining workers	0.82 (0.35–1.90)	1.11 (0.48–2.61)	2.50 (0.30–20.74)	3.37 (0.41–27.78)	0.80 (0.34–1.86)	1.07 (0.46–2.50)
6 Day-care workers	0.80 (0.30–2.10)	0.71 (0.28–1.80)	NA	NA	0.78 (0.30–2.06)	0.70 (0.28–1.77)
7 Dentists and dental workers	0.88 (0.18–4.45)	0.78 (0.16–3.79)	NA	NA	0.87 (0.17–4.36)	0.76 (0.15–3.79)
8 Drivers	1.33 (0.58–3.04)	1.79 (0.82–3.94)	5.42 (0.71–41.36)	**9.48 (1.14–78.92)**	1.30 (0.57–2.98)	1.76 (0.80–3.87)
9 Electrical and electronic production workers	0.66 (0.20–2.25)	0.83 (0.25–2.81)	NA	NA	0.65 (0.19–2.20)	0.82 (0.25–2.69)
10 Engine workshop workers	0.76 (0.23–2.50)	0.99 (0.28–3.58)	NA	NA	0.74 (0.23–2.45)	0.98 (0.28–3.47)
11 Farmers and agricultural workers	NA	NA	4.33 (0.87–21.70)	4.32 (0.84–22.29)	0.35 (0.09–1.28)	0.37 (0.10–1.42)
12 Forestry and related workers	NA	NA	**10.83 (1.67–70.19)**	**19.79 (3.23–121.32)**	0.87 (0.17–4.36)	1.26 (0.25–6.40)
13 Fur and leather workers	1.33 (0.49–3.62)	1.42 (0.46–4.35)	**8.13 (1.15–57.25)**	5.54 (0.76–40.33)	**1.95 (1.06–3.58)**	**2.06 (1.01–4.20)**
14 Glass, ceramic, and mineral workers	**2.65 (2.13–3.31)**	**3.96 (2.61–6.01)**	NA	NA	**2.60 (2.09–3.23)**	**3.82 (2.54–5.74)**
15 Hairdressers	0.53 (0.09–3.11)	0.59 (0.10–3.55)	NA	NA	0.52 (0.09–3.04)	0.56 (0.09 -3.43)
16 Housewives	0.53 (0.09–3.11)	0.48 (0.08–2.82)	NA	NA	0.52 (0.09–3.04)	0.46 (0.08–2.64)
17 Laboratory technicians	1.33 (0.33–5.40)	1.41 (0.49–4.03)	**16.25 (3.00–87.95)**	**30.66 (6.55–143.48)**	1.30 (0.32–5.29)	1.42 (0.50–4.05)
18 Metal workers	1.27 (0.80–2.03)	**1.84 (1.06–3.18)**	1.30 (0.15–11.15)	2.88 (0.34–24.33)	1.25 (0.79–1.98)	**1.80 (1.04–3.10)**
19 Maternity leave	NA	NA	NA	NA	NA	NA
20 Nurses and nursing associates	0.69 (0.35–1.35)	0.59 (0.30–1.15)	1.20 (0.14–10.35)	0.92 (0.11–7.58)	0.67 (0.34–1.32)	0.57 (0.29–1.12)
21 Painters	NA	NA	NA	NA	NA	NA
22 Physicians	1.33 (0.33–5.40)	1.68 (0.59–4.83)	NA	NA	1.30 (0.32–5.29)	1.59 (0.58–4.35)
23 Printers	NA	NA	NA	NA	NA	NA
24 Rubber and plastic workers	0.33 (0.05–2.10)	0.36 (0.05–2.41)	NA	NA	0.33 (0.05–2.06)	0.35 (0.05–2.38)
25 Retired	1.29 (0.87–1.91)	1.41 (0.90–2.20)	0.83 (0.10–7.24)	0.56 (0.06–4.87)	1.33 (0.92–1.94)	1.42 (0.93–2.19)
26 Sick leave	NA	NA	NA	NA	NA	NA
27 Storage workers	0.38 (0.06–2.36)	0.41 (0.07–2.35)	NA	NA	0.37 (0.06–2.31)	0.40 (0.07–2.34)
28 Students	0.80 (0.44–1.44)	0.82 (0.43–1.58)	1.08 (0.13–9.35)	0.91 (0.08–10.13)	0.78 (0.43–1.40)	0.78 (0.41–1.51)
29 Textile workers	0.59 (0.17–2.04)	0.54 (0.16–1.82)	NA	NA	0.58 (0.17–2.00)	0.52 (0.16–1.75)
30 Other occupations	1.19 (0.78–1.80)	1.22 (0.81–1.84)	NA	NA	1.16 (0.77–1.76)	1.18 (0.78–1.79)
31 Unemployed	0.77 (0.40–1.50)	0.80 (0.41–1.54)	1.35 (0.16–11.60)	1.37 (0.14–13.34)	0.76 (0.39–1.47)	0.78 (0.41–1.51)
32 Waiters	1.02 (0.50–2.10)	0.85 (0.41–1.74)	NA	NA	1.00 (0.49–2.06)	0.82 (0.40–1.68)
33 Wood and paper workers	0.59 (0.17–2.04)	0.69 (0.20–2.37)	NA	NA	0.58 (0.17–2.00)	0.68 (0.20–2.29)
Professionals, clerks, and administrative (REFERENCE)	REF	REF	REF	REF	REF	REF

*Abbreviations*: *CI* Confidence interval, *LRTI* Lower respiratory tract infection, *RR* Risk ratio

^a^Information on infections missing for altogether 12 participants: 3 professionals, clerks and administrative, 1 chemical industry worker, 1 cleaner, 2 metal workers, 2 retired, 1 unemployed, 2 with occupation missing

^b^Estimate for having at least one infection

^c^Participants with occupations that could not be included in the model (NA) were excluded from the analysis

^d^Adjusted for sex, age, and smoking

### Sensitivity analyses

Additional file 1: Supplementary Tables 1 and 2 present the crude and adjusted RR’s adjusted for three core covariates, three additional covariates, and all the six covariates. Most of the changes in the effect estimates were negligible (< 10%). Adjusting for ETS at home increased the effect estimate of chemical industry workers from 0.69 (0.13–3.57) to 1.00 (0.22–4.64) for URTI and from 1.21 (0.50–2.93) to 1.77 (0.91–3.43) for LRTI. The adjusted RR’s of forestry and related workers decreased from 1.22 to 1.13 and fur and leather workers increased from 2.06 (1.01–4.20) to 2.34 (1.22–4.45). In summary, the further adjustment did not influence the interpretation of the results.

## Discussion

The FEAS study was a population-based incident case–control study inviting all newly diagnosed cases of asthma among the working-age population within the study area in Southern Finland during the 2.5-year study period and randomly selected controls from the adult general population from the same area. Thus, the FEAS included a follow-up of 500,000 person-years, and the FEAS cases having incident asthma represent all new adult-onset asthma cases of the source population. In the present study we focused on the FEAS asthma case population to investigate if we can identify occupations that are related to an increased risk of catching respiratory infections, as this would provide important information for preventive actions and the results could be applied to asthma management among working-age populations. Thus, these results have clinical significance in asthma management, as well as more broadly public health significance in planning measures to prevent spreading of infections in work environments.

The susceptibility of subjects having asthma to catch respiratory infections has been shown in our recently reported study based on the Espoo Cohort Study from Finland, which reported that children and young adults having asthma experience increased risk of both URTIs with an adjusted incidence rate ratio (aIRR) 1.27 (95% CI 1.20 – 1.35) and LRTIs with aIRR 2.87 (95% CI 2.33–3.53) up to the age of 27 years [5]. Although the mechanisms for such susceptibility are not yet fully understood, both immunological dysfunction and structural airway alterations observed in subjects having asthma are likely to play a role.

The present study showed that occupations, where the workers often change their work environment according to the worksite that is under construction or is being otherwise worked on, for example in forestry, experienced particularly increased risk of common colds. Such workforces are rather mobile and may include workers from other parts of the same country or from other countries, which may spread infections especially during epidemics.

Increased risk of common colds was experienced by students, who may change their studying area frequently and attend lectures with a large audience being relatively close to each other, so their multiple contacts during the day may facilitate catching infections. In addition, those unemployed showed significantly increased unadjusted IRR of common cold, which raises the question whether a stressful situation in life increases susceptibility to catch respiratory infections. However, neither of these two occupations were linked to significantly increased risk when adjusted for potential confounders. In addition, those unemployed showed significantly increased unadjusted IRR of common cold, which raises the question whether a stressful situation in life increases susceptibility to catch respiratory infections.

Hairdressers and fur and leather workers experienced increased risks of tonsillitis and sinusitis, suggesting potential role of some chemicals used at work making them vulnerable to catch infections. Replacing such chemicals and other potentially irritating substances with less irritating ones might reduce infections in these occupations.

Laboratory technicians showed increased risk of tonsillitis, sinusitis as well as pneumonia, suggesting that close contact with the customers is a potential route of infections. Exposure to laboratory chemicals could also influence susceptibility of the airways to respiratory infections.

Increased risk of acute otitis media was detected among bakers and food processors, which raises the question whether this is due to frequent contacts with customers, or exposures related to preparation of food.

In contrast, increased risk of acute bronchitis was detected in occupations where workers often work in small workshops, in which they may work close to each other and where the ventilation may sometimes be insufficient. In addition, their work seemed to include handling of chemicals, such as glues among leather workers, or irritating substances, such as mineral fibers.

Increased risk of pneumonia was detected in occupations where the workers often work in close contact with the customers, such as laboratory technicians and drivers, the latter also sharing a rather small space with the customers. In such situations, it might be possible to prevent spreading of infections by increasing the volume of the work area and/or possibly by increasing air exchange rate in the work area.

Metal workers showed significantly increased risk of lower respiratory tract infections and borderline significantly increased risk of upper respiratory tract infections. It would be interesting to explore in the future, which features of metal work underlie the increased risk of respiratory infections detected in this study. Can exposure to metal dust increase susceptibility to infections or is exposure to metal working fluids or welding fumes underlying such susceptibility.

### Validity of the results

The study population was well defined because the diagnosis of incident asthma was based on reported asthma symptoms and objective findings of bronchial obstruction with significant bronchodilation effect in line with the national asthma guidelines applied at the time of the study period. Finland has had public health care since the early 1970s with easy access to a general practice physician. This is complemented by obligatory occupational health for adults at work. Both general practitioners and occupational health physicians can easily refer their patients to public hospitals having a Pulmonary Department, so the diagnosis of asthma is usually made at an early stage. In addition, also the fees by private practicing pulmonary physicians are partly reimbursed by the National Social Insurance. Thus, it is unlikely that the cases of newly diagnosed asthma would have had asthma for a long time before the diagnostic tests were performed.

The participation rate of cases of adult-onset asthma in the original population-based FEAS was good at 86%. All the information needed for the present analyses was available for 94.4% of the total FEAS asthma population. Thus, any major selection bias is unlikely in the present study.

Occupation at the time of diagnosis of asthma was the determinant of interest. The frequency and type of respiratory infections during the past 12 months formed the outcomes of interest. The outcomes were based on self-reporting in the FEAS questionnaire, which asked about both upper respiratory tract infections and lower respiratory tract infections in a systematic way. A limitation of this study is that we did not verify the infections with objective blood tests or microbial specimen. We considered confirming infections from the Finnish registry of health care visits (HILMO). However, this could have biased the results towards more severe respiratory infections, so we did not conduct this analysis. Those with asthma have been suggested to be more susceptible to experience respiratory infections in general [4, 5]. As this study was limited to those with recently diagnosed adult-onset asthma, comparison between different occupational groups can be considered valid, as although subjects in occupations related to increased rate of respiratory infections may later change their job due to this asthma-related susceptibility to infections, in the present study the majority of participants were still going through or had just gone through the diagnostic measurements for asthma when answering the FEAS questionnaire and the others had just recently received this diagnosis. Furthermore, the FEAS questionnaire also included a question inquiring if the study subject had previously changed or quit a job because of having asthma, and in the case of an affirmatory answer, they were asked to reply to the questions on respiratory infections based on the occupation from which they had changed to a new one.

In the multivariate regression analyses, we adjusted as potential confounders for age, gender, and smoking, all of which may be related to increased susceptibility to respiratory infections [9, 10]. Further, we conducted sensitivity analyses for URTI and LRTI by adding to the model with the three core covariates (age, gender and smoking), three additional potential confounders one at a time: having children at home, dampness and/or mold exposures at home, and tobacco smoke exposure at home and finally fitting all six covariates together. Most of the effect estimates remained stable (change < 10%) and none of the additional models influenced the interpretation of the results. This gives some assurance that the differences detected between occupations are related to the occupation or occupational environment linked to it.

The sample size in some of the occupations was small, which is reflected in wide confidence intervals. However, the lower 95% confidence limit was clearly above 1 for those occupations mentioned above. The original FEAS study was large including altogether 500 000 person-years, and the present analyses focused on the case population of FEAS to investigate if we can identify occupations that are related to an increased risk of catching respiratory infections, as this would provide important information for preventive actions and the results could be applied to asthma management for subjects with adult-onset asthma. Thus, these results have considerable clinical significance for asthma management as well as more broadly, public health significance for planning measures to prevent spreading of respiratory infections in workplaces.

### Synthesis with previous knowledge

Previous literature on the association between occupation and respiratory tract infections is still rather limited and focuses on the risk of influenza or influenza like illness [11–15]. The risk has been explained by different frequencies of contacts with other people, contact with contaminated surfaces at work or by work-related stress [15]. A recent register-based study found that people working in occupations with high person-to-person contact, such as work in day care, sewers, public transportation, and nursing and home care, had an increased risk of being hospitalized with pneumonia or influenza compared to people working within public administration [15]. These findings are in line with our results showing that drivers have increased risk of pneumonia and people working in day care centers have increased risk of URTIs. Furthermore, Pujol et al. suggested that manual workers, including people who are self-employed, workers in skilled technical occupations, workers qualified at the primary sector, and unskilled workers have a higher risk of hospitalization for influenza than people who are working in a non-manual occupation [14]. Consistent with our study, occupational groups related to food preparation and serving, community and social services, personal care and services, and building and ground cleaning as well as maintenance were associated with increased occurrence of influenza-like illness, defined as fever and cough or sore throat, in two questionnaire-based studies [11, 13].

Most of the previous studies were conducted in societies where access to health care services and the possibility to receive seasonal influenza vaccination could vary between different occupational groups. In Finland everyone with chronic diseases or working in health care has equal opportunity to receive vaccination within the national seasonal influenza vaccination program. Previous studies included general working aged populations, and some of them adjusted for co-morbidities, such as asthma, in the multivariate statistical models. However, none of the previous studies focused on a high-risk population as we did in our study that was based on a population of subjects with recently diagnosed adult-onset asthma.

## Conclusions

In this population-based Finnish Environment and Asthma Study of working-age adults with incident asthma from a geographically defined area in Southern Finland, the occupations that showed increased risk of common cold (as the major upper respiratory tract infection in this age group) were construction and mining and forestry and related workers. These are occupations with rather mobile workforces who change their work environment according to the need, and part of the workforce may live in shared accommodation close to the worksite. Such accommodations may include migratory workers who may have been infected with upper respiratory infections in other parts of the same country or worksites abroad. This study question is very topical because of the current pandemic with corona virus, i.e. SARS 2 virus that may have severe acute or long-term consequences for health. On the other hand, workers in occupations with often relatively small workspaces and exposure to some irritating substances, such as ceramic fibers or leather working materials, experienced increased occurrence of acute bronchitis and pneumonia, i.e. lower respiratory infections. Better understanding of the phenomena underlying these work-related risks would be useful for future prevention. Identifying the risk occupations for spreading of respiratory infections is useful for planning preventive strategies.

## Supplementary Information

Additional file 1: Supplementary Table 1. Risk of upper respiratory tract infections during past 12 months in people with newly-onset adult asthma according to the occupation in the FEAS. Supplementary Table 2. Risk of lower respiratory tract infections during past 12 months in people with newly-onset adult asthma according to the occupation in the FEAS.

## Data Availability

The datasets generated and/or analysed during the current study are not publicly available due to issues of confidentiality, but some data can be available from the corresponding author on reasonable request.

## Abbreviations

CI: Confidence interval
FEAS: The Finnish Environment and Asthma Study
IR: Incidence rate
IRR: Incidence rate ratio
LRTI: Lower respiratory tract infection
RTI: Respiratory tract infection
R: Risk ratio
RR: Risk ratio
URTI: Upper respiratory tract infection
