# Supplementary material for: Occupation and occurrence of respiratory infections among adults with newly diagnosed asthma
Source: BMC Pulm Med. 2023 Apr 25;23:140. doi: 10.1186/s12890-023-02413-8 (PMC10127176; doi:10.1186/s12890-023-02413-8)
Supplement: Supplementary file 1 — Additional file 1: Supplementary Table 1. Risk of upper respiratory tract infections during past 12 months in people with newly-onset adult asthma according to the occupation in the FEAS. Supplementary Table 2. Risk of lower respiratory tract infections during past 12 months in people with newly-onset adult asthma according to the occupation in the FEAS. [file 12890_2023_2413_MOESM1_ESM.docx]

**Supplementary table 1.** Risk of upper respiratory tract infections during past 12 months in people with newly-onset adult asthma according to the occupation in the FEAS.

|  | **URTI ^a, b, c^** | |  |  |  |  |
| --- | --- | --- | --- | --- | --- | --- |
|  | **Model 1** | | **Model 2**  **+ ETS home** | **Model 3**  **+ Visible mold or mold odor at home** | **Model 4**  **+ having children** | **Model 5**  **Saturated**  **+ ETS, mold odor at home, having children** |
| **Occupation** | **Crude RR**  **(95% CI)** | **Adjusted ^d, e^**  **RR (95% CI)** | **Adjusted ^d, e, f^**  **RR (95% CI)** | **Adjusted ^d, e^**  **RR (95% CI)** | **Adjusted ^d, e, g^**  **RR (95% CI)** | **Adjusted ^d, e, f, g^**  **RR (95% CI)** |
| 2 Bakers and food processors | 1.36  (0.56-3.32) | 1.46  (0.59-3.65) | 1.46  (0.58-3.65) | 1.49  (0.59-3.72) | 1.46  (0.59-3.66) | 1.48  (0.59-3.72) |
| 3 Chemical industry workers | 0.79  (0.14-4.41) | 0.69  (0.13-3.57) | 0.96  (0.22-4.22) | 0.70  (0.14- 3.65) | 0.71  (0.13-3.79) | 1.00  (0.22-4.64) |
| 4 Cleaners | 1.33  (0.82-2.16) | 1.26  (0.78-2.04) | 1.26  (0.78-2.05) | 1.27  (0.79-2.05) | 1.27  (0.78-2.05) | 1.28  (0.79-2.09) |
| 5 Construction and mining workers | 1.22  (0.59-2.54) | 1.67  (0.78-3.56) | 1.66  (0.78-3.54) | 1.64  (0.77-3.51) | 1.67  (0.78-3.58) | 1.63  (0.76-3.51) |
| 6 Day-care workers | **1.90**  **(1.08-3.35)** | 1.68  (0.96-2.94) | 1.67  (0.95-2.95) | 1.67  (0.95-2.94) | 1.67  (0.95-2.93) | 1.66  (0.94- 2.94) |
| 7 Dentists and dental workers | 1.06  (0.21-5.34) | 0.95  (0.19-4.71) | 0.95  (0.19-4.71) | 0.93  (0.20-4.33) | 0.95  (0.19-4.75) | 0.93  (0.20-4.31) |
| 8 Drivers | 1.06  (0.33-3.37) | 1.38  (0.39-4.80) | 1.37  (0.39-4.78) | 1.36  (0.40-4.63) | 1.36  (0.39-4.75) | 1.35  (0.39-4.59) |
| 9 Electrical and electronic production workers | 0.79  (0.23-2.70) | 0.98  (0.30-3.25) | 0.98  (0.30-3.27) | 0.97  (0.29-3.26) | 0.99  (0.30-3.24) | 0.96  (0.28-3.28) |
| 10 Engine workshop workers | 1.36  (0.56-3.32) | 1.52  (0.63-3.66) | 1.50  (0.62-3.63) | 1.50  (0.62-3.61) | 1.54  (0.64-3.68) | 1.50  (0.62-3.63) |
| 11 Farmers and agricultural workers | 1.27  (0.65-2.48) | 1.38  (0.70-2.71) | 1.44  (0.73-2.84) | 1.40  (0.71-2.75) | 1.39  (0.70-2.77) | 1.48  (0.75-2.93) |
| 12 Forestry and related workers | NA | NA | NA | NA | NA | NA |
| 13 Fur and leather workers | **2.38**  **(1.28-4.42)** | **2.87**  **(1.49-5.54)** | **2.90**  **(1.49-5.63)** | **2.90**  **(1.50-5.60)** | **2.90**  **(1.56-5.40)** | **2.97**  **(1.58-5.57)** |
| 14 Glass, ceramic, and mineral workers | NA | NA | NA | NA | NA | NA |
| 15 Hairdressers | 1.90  (0.89-4.06) | **2.14**  **(1.13-4.04)** | **2.14**  **(1.13-4.07)** | **2.11**  **(1.12-3.97)** | **2.19**  **(1.13-4.25)** | **2.15**  **(1.11-4.16)** |
| 16 Housewives | 1.27  (0.42-3.82) | 1.02  (0.33-3.10) | 1.02  (0.33-3.10) | 1.04  (0.34-3.19) | 1.00  (0.32-3.12) | 1.04  (0.33-3.26) |
| 17 Laboratory technicians | **3.17**  **(2.46 4.08)** | **2.87**  **(1.92-4.30)** | **2.86**  **(1.92-4.26)** | **2.93**  **(1.96-4.38)** | **2.87**  **(1.90-4.34)** | **2.91**  **(1.94-4.36)** |
| 18 Metal workers | 0.76  (0.36-1.60) | 0.98  (0.45-2.14) | 0.98  (0.45-2.13) | 0.99  (0.45-2.15) | 0.99  (0.45-2.16) | 0.98  (0.45-2.14) |
| 19 Maternity leave | **3.17**  **(2.46-4.08)** | **2.41**  **(1.79-3.24)** | **2.41**  **(1.78-3.25)** | **2.46**  **(1.82-3.34)** | **2.39**  **(1.77-3.24)** | **2.46**  **(1.79-3.38)** |
| 20 Nurses and nursing associates | 1.17  (0.68-2.04) | 1.03  (0.59-1.81) | 1.03  (0.59-1.81) | 1.02  (0.58-1.78) | 1.04  (0.59-1.81) | 1.02  (0.58-1.79) |
| 21 Painters | NA | NA | NA | NA | NA | NA |
| 22 Physicians | NA | NA | NA | NA | NA | NA |
| 23 Printers | 1.59  (0.39-6.49) | 1.51  (0.26-8.76) | 1.50  (0.26-8.67) | 1.54  (0.27-8.87) | 1.50  (0.26-8.67) | 1.53  (0.27-8.75) |
| 24 Rubber and plastic workers | 0.79  (0.23-2.70) | 0.86  (0.26-2.86) | 0.86  (0.26-2.85) | 0.88  (0.26-2.91) | 0.86  (0.26-2.85) | 0.87  (0.27-2.89) |
| 25 Retired | 1.14  (0.70-1.86) | 1.45  (0.86-2.45) | 1.51  (0.89-2.55) | 1.44  (0.85-2.44) | 1.45  (0.86-2.45) | 1.50  (0.89-2.54) |
| 26 Sick leave | NA | NA | NA | NA | NA | NA |
| 27 Storage workers | 0.45  (0.07-2.83) | 0.47  (0.07-3.09) | 0.47  (0.07-3.10) | 0.48  (0.07-3.14) | 0.47  (0.07-3.11) | 0.48  (0.07-3.15) |
| 28 Students | 1.27  (0.76-2.10) | 1.17  (0.65-2.10) | 1.16  (0.65-2.09) | 1.17  (0.65-2.11) | 1.17  (0.65-2.11) | 1.16  (0.64-2.10) |
| 29 Textile workers | 0.35  (0.06-2.27) | 0.36  (0.05-2.47) | 0.36  (0.05-2.48) | 0.36  (0.05-2.45) | 0.36  (0.05-2.47) | 0.36  (0.05-2.47) |
| 30 Other occupations | 1.00  (0.59-1.70) | 1.02  (0.61-1.72) | 0.96  (0.56-1.65) | 1.01  (0.60-1.69) | 1.02  (0.61-1.72) | 0.95  (0.55-1.62) |
| 31 Unemployed | 1.06  (0.57-1.96) | 1.07  (0.59-1.96) | 1.07  (0.58-1.95) | 1.07  (0.59-1.96) | 1.07  (0.59-1.96) | 1.07  (0.58-1.96) |
| 32 Waiters | 1.22  (0.59-2.54) | 0.99  (0.47-2.08) | 0.99  (0.47-2.08) | 1.01  (0.48-2.12) | 0.99  (0.47-2.07) | 1.00  (0.48-2.12) |
| 33 Wood and paper workers | 0.70  (0.20-2.45) | 0.77  (0.22-2.73) | 0.76  (0.21-2.71) | 0.78  (0.22-2.78) | 0.77  (0.22-2.74) | 0.78  (0.22-2.77) |
| Professionals, clerks, and administrative (REFERENCE) | REF | REF | REF | REF | REF | REF |

Abbreviations: CI, Confidence interval; LRTI, Lower respiratory tract infection; RR, Risk ratio

^a^ Information on infections missing for altogether 12 participants: 3 professionals, clerks and administrative, 1 chemical industry worker, 1 cleaner, 2 metal workers, 2 retired, 1 unemployed, 2 with occupation missing.

^b^ Estimate for having at least one infection.

^c^ Participants with occupations that could not be included in the model (NA) were excluded from the analysis.

^d^ Adjusted for sex, age, and smoking.

^e^ Information about smoking missing for 3 participants

^f^ Information about ETS at home during the past 12 months missing for 7 participants

^g^ Response to family related questions was missing from 41 participants, these were included in the analysis as a separate category of children

**Supplementary table 2.** Risk of lower respiratory tract infections during past 12 months in people with newly-onset adult asthma according to the occupation in the FEAS.

|  | **LRTI ^a, b, c^** | |  |  |  |  |
| --- | --- | --- | --- | --- | --- | --- |
|  | **Model 1** | | **Model 2**  **+ ETS home** | **Model 3**  **+ Visible mold or mold odor at home** | **Model 4**  **+ having children** | **Model 5**  **Saturated**  **+ ETS, molds at home, having children** |
| **Occupation** | **Crude RR**  **(95% CI)** | **Adjusted ^d, e,^**  **RR (95% CI)** | **Adjusted ^d, e, f^**  **RR (95% CI)** | **Adjusted ^d, e^**  **RR (95% CI)** | **Adjusted ^d, e, g^**  **RR (95% CI)** | **Adjusted ^d, e, f, g^**  **RR (95% CI)** |
| 2 Bakers and food processors | 0.37  (0.06-2.31) | 0.41  (0.06-2.59) | 0.41  (0.06-2.56) | 0.43  (0.07-2.67) | 0.41  (0.06-2.58) | 0.42  (0.07-2.63) |
| 3 Chemical industry workers | 1.30  (0.48-3.55) | 1.21  (0.50-2.93) | 1.60  (0.91-2.83) | 1.27  (0.52-3.10) | 1.25  (0.48-3.22) | 1.77  (0.91-3.43) |
| 4 Cleaners | 1.09  (0.68-1.74) | 0.96  (0.60-1.54) | 0.94  (0.59-1.50) | 0.98  (0.61-1.56) | 0.98  (0.61-1.57) | 0.98  (0.61-1.57) |
| 5 Construction and mining workers | 0.80  (0.34-1.86) | 1.07  (0.46-2.50) | 1.04  (0.45-2.44) | 1.03  (0.44-2.41) | 1.07  (0.45-2.54) | 1.00  (0.42-2.40) |
| 6 Day-care workers | 0.78  (0.30-2.06) | 0.70  (0.28-1.77) | 0.68  (0.27-1.72) | 0.69  (0.26-1.80) | 0.69  (0.28-1.75) | 0.67  (0.26-1.73) |
| 7 Dentists and dental workers | 0.87  (0.17-4.36) | 0.76  (0.15-3.79) | 0.75  (0.15-3.70) | 0.72  (0.13-3.96) | 0.75  (0.15-3.80) | 0.70  (0.13-3.90) |
| 8 Drivers | 1.30  (0.57-2.98) | 1.76  (0.80-3.87) | 1.72  (0.78-3.80) | 1.74  (0.81-3.72) | 1.75  (0.79-3.87) | 1.69  (0.78-3.69) |
| 9 Electrical and electronic production workers | 0.65  (0.19-2.20) | 0.82  (0.25-2.69) | 0.86  (0.25-2.96) | 0.79  (0.23-2.67) | 0.80  (0.24-2.64) | 0.82  (0.23-2.86) |
| 10 Engine workshop workers | 0.74  (0.23-2.45) | 0.98  (0.28-3.47) | 0.95  (0.27-3.41) | 0.95  (0.28-3.30) | 1.03  (0.29-3.64) | 0.98  (0.28-3.44) |
| 11 Farmers and agricultural workers | 0.35  (0.09-1.28) | 0.37  (0.10-1.42) | 0.40  (0.10-1.51) | 0.38  (0.10-1.46) | 0.39  (0.10-1.49) | 0.42  (0.11-1.61) |
| 12 Forestry and related workers | 0.87  (0.17-4.36) | 1.26  (0.25-6.40) | 1.22  (0.24-6.19) | 1.18  (0.21-6.55) | 1.25  (0.25-6.31) | 1.13  (0.21-6.27) |
| 13 Fur and leather workers | **1.95**  **(1.06-3.58)** | **2.06**  **(1.01-4.20)** | **2.18**  **(1.20-3.98)** | **2.11**  **(1.03-4.30)** | **2.15**  **(1.01-4.56)** | **2.34**  **(1.22-4.45)** |
| 14 Glass, ceramic, and mineral workers | **2.60**  **(2.09-3.23)** | **3.82**  **(2.54-5.74)** | **3.78**  **(2.53-5.65)** | **3.95**  **(2.63-5.95)** | **3.85**  **(2.49-5.94)** | **3.98**  **(2.58-6.14)** |
| 15 Hairdressers | 0.52  (0.09-3.04) | 0.56  (0.09 -3.43) | 0.55  (0.09-3.38) | 0.55  (0.09-3.42) | 0.59  (0.10-3.48) | 0.56  (0.09-3.41) |
| 16 Housewives | 0.52  (0.09-3.04) | 0.46  (0.08-2.64) | 0.45  (0.08-2.61) | 0.48  (0.08-2.78) | 0.47  (0.08-2.72) | 0.49  (0.08-2.89) |
| 17 Laboratory technicians | 1.30  (0.32-5.29) | 1.42  (0.50-4.05) | 1.39  (0.49-3.94) | 1.49  (0.52-4.30) | 1.41  (0.48-4.09) | 1.44  (0.48-4.28) |
| 18 Metal workers | 1.25  (0.79-1.98) | **1.80**  **(1.04-3.10)** | **1.82**  **(1.06-3.12)** | **1.83**  **(1.07-3.13)** | **1.80**  **(1.04-3.12)** | **1.84**  **(1.07-3.15)** |
| 19 Maternity leave | NA | NA | NA | NA | NA | NA |
| 20 Nurses and nursing associates | 0.67  (0.34-1.32) | 0.57  (0.29-1.12) | 0.57  (0.29-1.11) | 0.56  (0.28-1.10) | 0.58  (0.30-1.12) | 0.56  (0.28-1.10) |
| 21 Painters | NA | NA | NA | NA | NA | NA |
| 22 Physicians | 1.30  (0.32-5.29) | 1.59  (0.58-4.35) | 1.57  (0.57-4.32) | 1.60  (0.58-4.42) | 1.57  (0.57-4.35) | 1.59  (0.57-4.42) |
| 23 Printers | NA | NA | NA | NA | NA | NA |
| 24 Rubber and plastic workers | 0.33  (0.05-2.06) | 0.35  (0.05-2.38) | 0.34  (0.05-2.30) | 0.36  (0.05-2.45) | 0.35  (0.05-2.33) | 0.35  (0.05-2.34) |
| 25 Retired | 1.33  (0.92-1.94) | 1.42  (0.93-2.19) | 1.45  (0.95-2.23) | 1.42  (0.93-2.16) | 1.42  (0.93-2.17) | 1.45  (0.95-2.20) |
| 26 Sick leave | NA | NA | NA | NA | NA | NA |
| 27 Storage workers | 0.37  (0.06-2.31) | 0.40  (0.07-2.34) | 0.39  (0.07-2.29) | 0.42  (0.07-2.44) | 0.40  (0.07-2.36) | 0.41  (0.07-2.41) |
| 28 Students | 0.78  (0.43-1.40) | 0.78  (0.41-1.51) | 0.78  (0.40-1.50) | 0.78  (0.41-1.50) | 0.78  (0.41-1.50) | 0.77  (0.40-1.49) |
| 29 Textile workers | 0.58  (0.17-2.00) | 0.52  (0.16-1.75) | 0.50  (0.15-1.70) | 0.51  (0.15-1.67) | 0.53  (0.16-1.76) | 0.50  (0.15-1.64) |
| 30 Other occupations | 1.16  (0.77-1.76) | 1.18  (0.78-1.79) | 1.13  (0.74-1.73) | 1.15  (0.75-1.75) | 1.18  (0.79-1.78) | 1.09  (0.71-1.69) |
| 31 Unemployed | 0.76  (0.39-1.47) | 0.78  (0.41-1.51) | 0.78  (0.40-1.49) | 0.79  (0.42-1.48) | 0.79  (0.41-1.51) | 0.78  (0.42-1.47) |
| 32 Waiters | 1.00  (0.49-2.06) | 0.82  (0.40-1.68) | 0.80  (0.39-1.63) | 0.85  (0.41-1.76) | 0.82  (0.40-1.68) | 0.83  (0.40-1.71) |
| 33 Wood and paper workers | 0.58  (0.17-2.00) | 0.68  (0.20-2.29) | 0.69  (0.21-2.30) | 0.71  (0.21-2.39) | 0.68  (0.20-2.29) | 0.72  (0.22-2.38) |
| Professionals, clerks, and administrative (REFERENCE) | REF | REF | REF | REF | REF | REF |

Abbreviations: CI, Confidence interval; LRTI, Lower respiratory tract infection; RR, Risk ratio

^a^ Information on infections missing for altogether 12 participants: 3 professionals, clerks and administrative, 1 chemical industry worker, 1 cleaner, 2 metal workers, 2 retired, 1 unemployed, 2 with occupation missing.

^b^ Estimate for having at least one infection.

^c^ Participants with occupations that could not be included in the model (NA) were excluded from the analysis.

^d^ Adjusted for sex, age, and smoking.

^e^ Information about smoking missing for 3 participants

^f^ Information about ETS at home during the past 12 months missing for 7 participants

^g^ Response to family related questions was missing from 41 participants, these were included in the analysis as a separate category of having children
